# Immunology of Infectious Diseases

**DOI:** 10.3201/eid0811.020430

**Published:** 2002-11

**Authors:** Thomas Lazar

**Affiliations:** Institute of Transfusion Medicine, Humboldt University Medical School, Berlin, Germany

Whether an infectious disease agent is an “old acquaintance” or a new, emerging threat, the immune system’s battle against it is usually the first line of defense it encounters. With vaccines and effective treatments often unavailable, the immune system’s efforts to eradicate infectious agents or infected cells are frequently the only means to combat them. Understanding the immune system—as well as the infectious agent’s tactics to undermine it—is of vital importance to the researcher and clinician. This textbook attempts to provide just this information. 

Immunology of Infectious Diseases is a textbook in the best sense of the word, presenting its contents in a clear, structured manner. Instead of encylopedic coverage of every infectious disease agent known, a set of paradigmatic infections were selected on the basis of the depth of available knowledge. The book is divided into eight sections, each of which addresses a particular aspect of the host-infectious agent interaction, describing it in separate chapters for bacteria, fungi, parasitic eukaryotes, and viruses. So instead of discussing all aspects of viral diseases, the reader learns about the innate immune response to the various pathogens, chapter by chapter, in the respective section. Emphasis is thereby placed on the immune system’s “point of view” about an infectious process, rather than on the microbe’s. 

After an introduction to the various classes of infectious disease agents, the book describes the immune responses directed against the different types of infections, proceeding from the innate to the acquired (adaptive). Discussion of the pathology of infections not eradicated by the immune system early on and the cunning strategies of the infectious microbes to evade immune attacks is followed by sections on immunogenetics and exploration of the immune system’s interventions against two high-incidence infections, tuberculosis and AIDS. 

Although the infections discussed in this book are not emerging ones in the strictest sense, the example of AIDS shows just how fast an infectious disease that was emerging, seemingly restricted to a subset of the population only two decades ago, can grow into a pandemic in a highly mobile, dense population at the end of the 20th century. Even tuberculosis, the “wasting disease” dreaded by our grandfathers’ generation, which scientists believed to be under control, can be regarded as an emerging disease:* Mycobacterium tuberculosis* has stepped into the limelight again in the wake of HIV, which renders a growing number of people immunocompromised. 

As infection and immune reaction are so intricately intertwined, this book is valuable reading to anyone interested in infectious diseases in humans. Maybe in the future prions will have to be included as a new type of infectious agent whose rise we are just now witnessing. (Information on prion pathology is still hotly debated, and data on routes of transmission and immune system reactions are still scarce.) 

The book is a handy and manageable length. In contrast to many standard textbooks of immunology, Immunology of Infectious Diseases is text oriented. Except for a central insert of color plates, figures are in black and white only. Each chapter contains an alphabetical list of references. Because of its accessible modular structure, this textbook is easy to navigate, rendering it easy to use. The book is suitable for anyone with a background in cell biology and basic immunology. Advanced undergraduate students and postgraduates with a grasp of the main groups of leukocytes and immune effector mechanisms, as well as specialists in other subdisciplines, will find this textbook to be a highly useful and readable introduction to the immune system’s mainstay, the battle against infection.

